# Infective or Non-Infective Endocarditis: A Brief Literature Review Based on a Case Report

**DOI:** 10.3390/jcm14082675

**Published:** 2025-04-14

**Authors:** Vasiliki Tsolaki, George E. Zakynthinos, Konstantina Deskata, Ilias Dimeas, Kyriaki Parisi, Athanasia Makrygianni, Grigorios Giamouzis, Epaminondas Zakynthinos, Andrew Xanthopoulos

**Affiliations:** 1 Critical Care Department, University Hospital of Larissa, Faculty of Medicine, University of Thessaly, Mezourlo, 41335 Larissa, Greece; vasotsolaki@yahoo.com (V.T.); konstadv@gmail.com (K.D.); kyriakiparisi@yahoo.com (K.P.); athama1001@hotmail.com (A.M.); 2 3rd Department of Cardiology, “Sotiria” Chest Diseases Hospital, Medical School, National and Kapodistrian University of Athens, 11527 Athens, Greece; ezakynth@yahoo.com; 3 Respiratory Department, University Hospital of Larissa, Faculty of Medicine, University of Thessaly, 41110 Larissa, Greece; dimel13@hotmail.com; 4 Department of Cardiology, University Hospital of Larissa, Faculty of Medicine, University of Thessaly, 41110 Larissa, Greece; grgiamouzis@gmail.com (G.G.); andrewvxanth@gmail.com (A.X.)

**Keywords:** influenza, nonbacterial thrombotic endocarditis, hypercoagulable state, vegetations

## Abstract

In the present report, we describe a patient presenting in the intensive care unit with fever, respiratory failure, and multiple lesions on cardiac valves. The patient, with a history of multiple myeloma under treatment, was intubated due to ARDS from influenza, and cardiac ultrasonography revealed lesions in the aortic, mitral, and tricuspid valves. There is a step-by-step approach in the case presentation, with clinical questions, while there is a review of the current literature concerning the issue.

## 1. Introduction

Influenza A infection commonly affects the upper respiratory tract, resulting in a self-limited infection with mild respiratory symptoms. In severe cases, which typically occur in elderly or immunocompromised patients, influenza may lead to viral pneumonia, progressing to acute lung injury, a syndrome of increased pulmonary microvascular leakage leading to pulmonary edema, hypoxemia, and respiratory failure. Following the release of inflammatory cytokines, the flu syndrome can favor the triggering of a prothrombotic state, facilitating platelet activation and endothelial dysfunction. Acute influenza infections are linked to an increased risk of developing not only venous but also arterial thromboembolic events [1,2,3]. The pathogenesis of arterial thrombosis, differing from that of venous thrombosis, typically occurs as a result of endovascular injury or endothelial activation caused by the elaboration of a variety of proinflammatory mediators with subsequent platelet and coagulation pathway activation [4]. Thus, the presence of both venous and arterial thrombosis during influenza may co-exist [4].

Nonbacterial thrombotic endocarditis (NBTE) is a rare condition characterized by the formation of sterile vegetations on the surface of heart valves. NBTE occurs most commonly in association with malignancies, autoimmune conditions (antiphospholipid antibody syndrome, systemic lupus erythematosus), and has been reported in association with burns, sepsis, and indwelling catheters and SARS-CoV-2 infection [5,6]. However, an interplay between endothelial injury, hypercoagulability, hypoxia, and immune complex deposition appears to be responsible for the formation of sterile valvular vegetations in NBTE [5].

In patients with influenza ARDS presenting with mass lesions in heart valves, nonbacterial thrombotic endocarditis should be suspected. In the present report, we describe a patient presenting in the intensive care unit with fever, respiratory failure due to influenza ARDS, and multiple lesions on cardiac valves. There is a step-by-step approach in the case presentation, with clinical questions giving an educational character to the approach, while there is review of the current literature concerning the issue.

## 2. Case Presentation

A 52-year-old female patient (BMI: 23 kg/m^2^, non-smoker) was admitted in the intensive care unit (ICU) with acute respiratory distress syndrome (ARDS). Her medical history involved multiple myeloma (autologous hematopoietic cell transplantation 6 months previously, currently under immunosuppressive therapy with recent assessment of complete response) and had been immunized for the current influenza season. At present, she complained of fever, non-productive cough, and worsening dyspnea during the previous seven days. Upon presentation in the emergency department (ED), she was hypoxemic [PaO_2_: 41 mmHg (FiO_2_: 21%)], and her labs included the following: white blood cells (WBC) 2000 cells/μL and C-reactive protein (CRP) 20 mg/dL (normal value < 0.5 mg/dL), lactic acid: 2.8 mmol/lt. Her chest X-ray revealed bilateral alveolar infiltrates. Blood cultures were drawn, and the patient was initially admitted to the hematology department and was started on antimicrobial therapy that included moxifloxacin, linezolid, meropenem, oseltamivir, and trimethoprim sulphamethoxazole, while an oropharyngeal swab was sent for the detection of various pathogens (polymerase chain reaction for viruses and community pathogens) and turned positive for influenza type A (H1N1). Urine Pneumococcus and Legionella Ag were negative. Two days later, the patient’s oxygenation status deteriorated, and she was transferred to the ICU.

On arrival, she was in respiratory distress (RR: 40/min), with hypoxemia (PaO_2_/FiO_2_: 115 mmHg) but remained hemodynamically stable. Auscultation revealed diffuse rales on both lungs and a diastolic murmur indicating aortic insufficiency. Her chest X-ray revealed diffuse alveolar infiltrates bilaterally. Laboratory tests revealed hemoglobin of 9.9 g/dL, WBC: 4700/μL, platelets (PLT) 113,000/μL; coagulation and liver function tests were normal [total bilirubin 0.28 mg/dL, PT: 1.21, aPTT: 37.9 fibrinogen was 479 mg/dL (180–440 mg/dL), LDH was 1288 IU/L].

**Figure 1:**
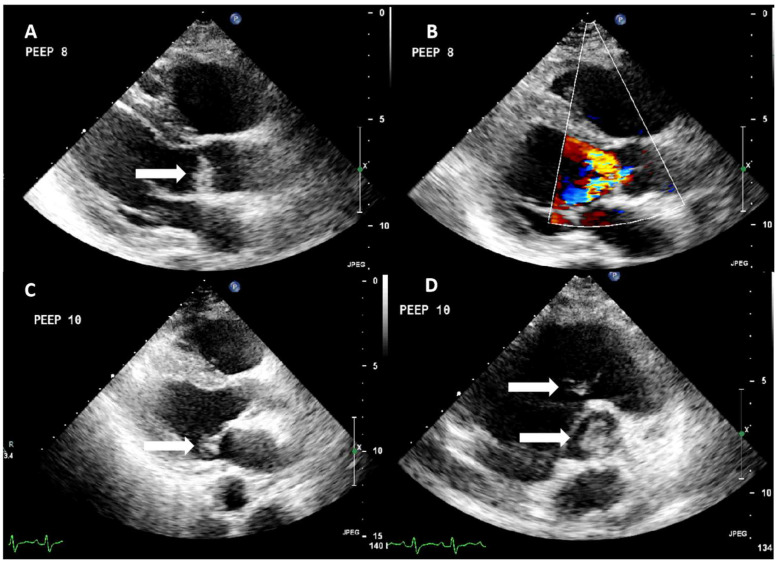
Echocardiographic findings presenting lesions in multiple valves. (**A**,**B**) Parasternal long-axis view showing a mass (vegetation) attached to the ventricular surface of the aortic valve (indicated by the white arrow). There is significant aortic regurgitation with two eccentric jets. (**C**) Parasternal long-axis view showing a mass attached to the atrial surface of mitral valve (white arrow). (**D**) Modified parasternal short-axis view focusing on the right chambers, showing a thin vegetation attached to the ventricular surface and the subvalvular apparatus of the tricuspid valve (white arrow). There is also vegetation attached to the aortic valve (white arrow).

Soon after ICU admission, oxygenation status further worsened, and the patient was finally intubated. To further evaluate the reason for aortic insufficiency and general systolic/diastolic cardiac function, bedside echocardiography was performed, revealing a mass attached to the downstream side of the left coronary cusp of the aortic valve (Figure 1). Severe aortic insufficiency with two eccentric jets were visualized (Figure 1, Videos S1 and S2).

There was also a smaller lesion attached to the posterior leaflet of the mitral valve and a mobile mass in the right ventricle attached to the tricuspid valve (Videos S3 and S4 and Figure 1). As the findings strongly indicated the presence of possible infective endocarditis, gentamycin was added to the patient’s antimicrobial regime (which included meropenem and vancomycin instead of linezolide, trimethoprime-sulphamethoxazole, moxifloxacin, and oseltamivir).

Tracheobronchial aspirate culture turned negative; in addition, the urine antigens for Legionella and Streptococcus pneumonia were negative (a repeat of the examination after ICU admission). The blood cultures (incubation for 21 days) turned negative, as did the serology tests for Coxiella burnetii and mycoplasma.

**Figure 2:**
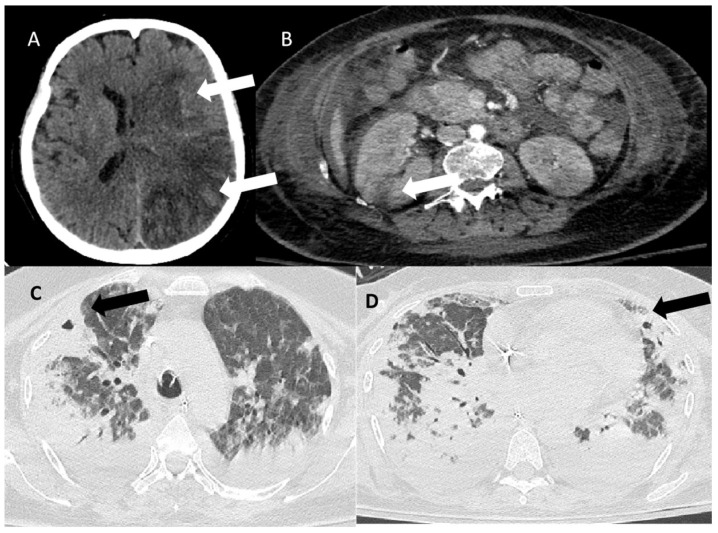
Computed tomography findings. (**A**) Brain CT showing multiple ischemic lesions located in the left frontotemporolateral region (white arrow) and in the right frontal lobe. Effacement of the left lateral ventricle and displacement of the midline structures by 0.5 cm to the right. Trace of subarachnoid hemorrhage (white arrow). (**B**) Ischemic infarct at the periphery of the right kidney (right arrow). (**C**) Cavitary lesions in the right upper and (**D**) left lower lobe (black arrows).

On the 10th day, the patient presented signs of right hemiparesis, and the results of the brain CT are presented in Figure 2. In Figure 2, images of the chest CT are also displayed.

### What Is the Diagnosis?

Diagnosis: Nonbacterial thrombotic endocarditis possibly triggered by influenza infection in an immunocompromised patient with multiple myeloma, complicated by systemic and pulmonary embolization.

## 3. Discussion

Here, we present a patient with ARDS due to influenza infection and NBTE in multiple valves, with subsequent multiple embolic episodes. To our knowledge, only one patient with NBTE affecting multiple valves, suffering embolic episodes, in the setting of an H1N1 infection, and also presenting with ARDS, has ever been reported [7]. Both patients presented NBTE in multiple valves and suffered embolic episodes during hospitalization for the index infection.

Nonbacterial thrombotic endocarditis (NBTE), is a form of noninfectious endocarditis characterized by the deposition of sterile platelet thrombi on heart valves, commonly affecting undamaged valves [8]. It occurs in patients with a predisposing factor and/or a hypercoagulable state, such as cancer (marantic endocarditis), systemic lupus erythematous (SLE), catastrophic antiphospholid syndrome (Libman–Sacks endocarditis), disseminated intravascular coagulation (DIC), or other chronic diseases (tuberculosis or autoimmune disease) [6,8]. Although the exact pathogenetic mechanism of NBTE is unknown, several factors are thought to contribute to lesion formation, including endothelial dysfunction, hypercoagulability, and immune complex disposition [5,9]. Increased production of coagulation factors, cytokines, and high tissue factor expression are potential mechanisms underlying NBTE in cancer patients. NBTE probably arises from endothelial injury caused by circulating cytokines during the hypercoagulable condition [10]. Hypoxia is also associated with the formation of NBTE. A rat model of hypoxia at a level of a 5500 m high-altitude environment showed a 100% rate of NBTE after 12 weeks of exposure in such conditions. There was increased expression of prothrombotic tissue factor in animals kept in hypoxic conditions compared to control normoxic animals with normal valves [11]. In an autopsy study in 50 patients with NBTE, the lung weight was greater compared to controls, and there were also clinical and histopathological evidence of more severe pulmonary disease compared to matched controls. Only three patients with NBTE did not have any lung pathology in autopsy studies [12]. These results stress the association of hypoxia-induced hypercoagulable conditions with NBTE formation.

Contrary to infectious endocarditis, the vegetations in NBTE are not of inflammatory nature and can be classified as fibrin-rich ‘white thrombi’. They are often only loosely connected to the heart valves as there is no inflammatory reaction at the site of attachment, which in turn precipitates dislodgement with the following embolization. Thus, in NBTE there is a greater tendency for vegetations to embolize and cause extensive infarction. Systemic embolization occurs in 40% of the patients, mainly in the central nervous system, kidneys, extremities, spleen, and coronary arteries, as in our patient [8,13].

Patients with NBTE are typically asymptomatic until embolization occurs. A high index of clinical suspicion is warranted. There are no laboratory tests to confirm the diagnosis of NBTE, which is based on exclusion of others, mainly infective endocarditis. NBTE should be suspected in patients with acute stroke or multiple widely distributed emboli. A workup for a hypercoagulable state (in particular, testing for the presence of a lupus anticoagulant and elevated levels of antiphospholipid antibodies) and disseminated intravascular coagulation (DIC) should be performed in every case of suspected NBTE [13]. Transthoracic and transesophageal echocardiography are important diagnostic modalities in the work-up of patients with suspected NBTE. Diffuse thickening of the affected leaflet is the most common finding. Vegetations are frequently left-sided but multivalvular involvement has been reported, yet not in influenza infections [8,13]. Moreover, NBTE vegetations do not alter the valve morphology, although aortic insufficiency was seen in the presented patient [8]. Concerning the treatment, the 2023 European Society of Cardiology (ESC) guidelines for endocarditis underline the importance of anticoagulation as the mainstay of treatment in patients with NBTE secondary to the risk of recurrent embolization and the fragile nature of the thrombi [8]. The presented patient could not receive anticoagulation due to persistent thrombocytopenia attributed to liver failure. Another treatment option in NBTE includes surgical intervention in the case of persistent embolic events despite medical therapy and, less commonly, heart failure and acute valve rupture. Indication for surgery should be based on the guidelines for conventional infective endocarditis. Naturally, treatment of the underlying cause of NBTE is necessary, if one can be identified. Treatment of the underlying condition and anticoagulation, if not contraindicated, should be initiated [5,13,14].

Influenza A virus is a respiratory pathogen that substantially affects human health worldwide. The main complication of influenza infection is viral pneumonia, which often occurs together with, or is followed by, bacterial pneumonia, mainly due to staphylococcus or streptococcus superinfection. Acute respiratory distress syndrome (ARDS) is one of the most important complications with an increased mortality rate [15]. ARDS involves alveolar damage to the epithelial–endothelial barrier, fluid leakage into the alveolar lumen, and respiratory insufficiency. The most important part of the epithelial–endothelial barrier is the alveolar epithelium, strengthened by tight junctions. The influenza virus targets these epithelial cells, reducing sodium pump activity, damaging tight junctions, and killing infected cells. Infected epithelial cells produce cytokines that attract leucocytes—neutrophils and macrophages—and activate adjacent endothelial cells [15]. This process is followed by the elaboration of a variety of proinflammatory mediators with subsequent platelet and coagulation pathway activation, in conjunction with alterations in the balance of procoagulant and anticoagulant factors [16]. In more detail, on endothelial injury, platelets are recruited by inflamed endothelial cells, where they adhere and are activated [17]. Importantly, platelet activation is strongly associated with enhanced inflammatory responses. Activated platelets release potent inflammatory molecules and play a key role in leukocyte recruitment. It has been shown that endothelial cell infection by the influenza virus can trigger apoptosis, stimulating platelet adhesion [17,18]. Patients with ARDS due to A(H1N1) had increased platelet activation compared with bacterial pneumonia patients of similar age, sex, and APACHE II scores [19]. Moreover, the influenza virus may activate platelets and generate thrombin [20].

Influenza was not identified as an independent risk factor in a case–control study of patients suspected of having pulmonary embolism [2]. Conversely, in a recent case presentation and review of the literature concerning clinical characteristics of patients suffering a thrombotic episode during the course of influenza infection, 36.2% of the cases had “de novo” pulmonary embolism with no evidence of underlying deep venous thrombosis. “De novo” pulmonary embolism in the general population ranges from 0 to 22% [4]. Local inflammation of the lung parenchyma may theoretically contribute to endothelial activation and the subsequent induction of a hypercoagulable state in the surrounding microenvironment [4]. Additionally, during influenza virus infection, the extrinsic coagulation pathway was stimulated with a reduced generation of key inhibitors of coagulation and fibrinolysis, namely activated protein C and plasminogen—activator inhibitor type-1 [21]. This may also lead to distant thrombosis. In a recent observational, registry-based, self-controlled case series study evaluating the association between laboratory-confirmed influenza infection and the occurrence of acute myocardial infarction, the former was associated with an increased risk of acute myocardial infarction, especially in individuals without a prior hospitalization for coronary artery disease [1]. Moreover, among 24,103 cases of 18 to 44 year olds, the stroke risk 30 days after an influenza-like illness was increased (aOR, 1.68 [95% CI, 1.51–1.86]) [22]. All these mechanisms suggest a hypercoagulable state during the course of an influenza infection, which, together with epithelial injury induced by the circulating cytokines may lead to nonbacterial thrombotic endocarditis.

### Clinical Course

The presence of lesions in heart valves indicated endocarditis, and the working diagnosis was infective endocarditis due to a bacterial super-infection in the lungs, in an immunocompromised patient with influenza. Yet, all blood cultures drawn upon hospital (before the receipt of any antimicrobial agent) and ICU admission and the tracheobronchial aspirate cultures did not reveal any pathogen apart from influenza. The presence of pneumococcal and Legionella antigens in the urine were negative twice and so were the serology examinations for *Coxiella burnetti* and mycoplasma. Taking these laboratory results into consideration, infective endocarditis was essentially excluded. Moreover, infective endocarditis vegetations are usually larger and the embolic risk corresponds to the size, driving the decision for surgical management [8]. Reviewing the patients’ medical records, echocardiography performed three months before the index hospitalization was unremarkable; so, myxomatous valve disease could not be supported. Thus, NBTE was suspected. Concerning the contributing factors that led to NBTE, the investigation for rheumatic diseases (antinuclear antibodies, rheumatoid factor, anticardiolipin antibodies) was unremarkable, and multiple myeloma had not relapsed (absence of monoclonal protein in serum and urine by immunofixation).

To our knowledge, infective endocarditis due to influenza infection has scarcely been reported. In fact, we identified only one report of a patient with influenza ARDS and multiple valve lesions, leading to cerebral infarction. Certainly, the NBTE diagnosis in our patient was made after exclusion of other possible conditions, as no biopsy of the embolic lesions was performed. Thus, a definitive diagnosis cannot be established. Yet, we believe that multivalvular involvement, the negative blood cultures (even the cultures collected before administration of any antibiotic), and the early occurrence of multiple embolic episodes (suggesting the frailty of vegetations) support the diagnosis of NBTE. Cavitary lesions identified in the chest CT are located in the periphery of the lung parenchyma, and the characteristic appearance (triangular lesion with the base attached to the pleura) indicates an embolic origin of the lesion. Pulmonary embolic complication in a patient with ARDS might have occurred from local thrombosis as a result of parenchymal inflammation. Yet, embolic lesions affecting other organs as well (brain, kidney) support a more central (cardiac) origin of the embolic burden. Multiple myeloma and a hypercoagulable state induced by the influenza infection may have triggered the vegetation formation. Importantly, it was the acute hypercoagulable state (influenza infection) added to a chronic inflammatory condition (multiple myeloma) that induced vegetation formation.

**Figure 3:**
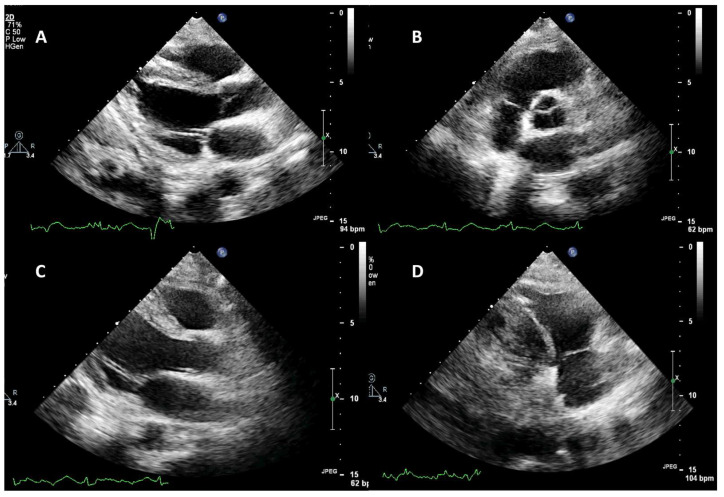
Echocardiographic evaluation of cardiac valves on the 20th ICU day, after the patient suffered the embolic episodes. (**A**,**B**) Parasternal long-axis and short-axis view of the aortic valve. There is no residual pathology indicating the initial vegetation. (**A**,**C**) Parasternal long-axis view of the mitral valve. No pathology is identified. (**D**) Modified parasternal long-axis view focusing on the right chambers. There is no lesion attached to the tricuspid valve.

Repeat heart ultrasonography demonstrated complete resolution of the lesions attached on the aortic, mitral, and tricuspid valves (Figure 3). Transesophageal echocardiography (TEE) was performed on the 50th day (on thrombocytopenia resolution) revealing the resolution of the valvular vegetations and aortic insufficiency of a mild degree (Figure 3). The patient was weaned from the ventilator on the 60th day with a Glasgow coma scale of 15 and was discharged to a long-term health care center and ultimately weaned from the artificial airway.

## 4. Conclusions

In conclusion, influenza infection induces a hypercoagulable state, and in the setting of hypoxia, such as in ARDS, the thrombotic risk may be exaggerated. We believe that patients with ARDS due to influenza infection should undergo an extensive evaluation with echocardiography, as the coexistence of NBTE may be more prevalent. The case presented here developed NBTE in the setting of influenza ARDS and had multiple valve lesions, which, being very friable, resulted in embolic complication. Systematic evaluation of the cardiac valves in patients with influenza ARDS will depict the true burden of NBTE in these patients.

## 5. Clinical Pearls

1. Influenza infection may induce a hypercoagulable state in the infected patients. Practitioners should keep that in mind so that the patients receive proper anticoagulation.
2. Influenza infection activates platelets, fueling inflammation. Patients with influenza-induced infections, presenting signs incompatible with their initial diagnosis, should be further evaluated for additional pathologies. Cardiac ultrasound may be valuable in the evaluation of patients with influenza ARDS.
3. Patients with unexplained embolic episodes, during the course of other illnesses, should have a thorough evaluation for the presence of nonbacterial thrombotic endocarditis.

## Supplementary Materials

The following supporting information can be downloaded at: https://www.mdpi.com/article/10.3390/jcm14082675/s1, Video S1: Parasternal long-axis view showing the extra tissue attached to the ventricular surface of the aortic valve and an oscillating structure seen in the right ventricle. Video S2: Parasternal long-axis view showing a mass (vegetation) attached to the ventricular surface of the aortic valve. There is significant aortic regurgitation with two eccentric jets. Videos S3 and S4: Modified parasternal short-axis view focusing on the right chambers, showing a mobile vegetation attached to the ventricular surface and the subvalvular apparatus of the tricuspid valve. The vegetation of the aortic valve is also seen from this view. Video S5: Parasternal long-axis view. Clear aortic valve (there is no pathology identified). Video S6: parasternal short-axis view. Clear aortic valve (there is no pathology identified). There is no abnormal finding at the level of the tricuspid valve.
